# School Reporting of a Dengue Outbreak — St. Croix, U.S. Virgin Islands, 2012

**Published:** 2013-03-08

**Authors:** Thomas Morris, Francine Lang, Romeo Christopher, Darice Plaskett, Brad Biggerstaff, Kalanthe Horiuchi, George Han, Brett Ellis, Manuel Amador, Gilberto Felix, Manuela Beltran, Kay Tomashek, Jorge Muñoz-Jordan, Elizabeth Hunsperger, Roberto Barrera, Harold Margolis, Dana Thomas, Esther Ellis

**Affiliations:** Virgin Islands Dept of Health; Div of Vector-Borne Diseases; Dengue Br, Div of Vector-Borne Diseases, National Center for Emerging and Zoonotic Infectious Diseases; EIS officers, CDC

Dengue is endemic in the U.S. Virgin Islands, but no outbreaks have been reported since 2005 (1). In November 2012, a school nurse in St. Croix reported suspected dengue in 27 (7%) of 369 students and staff members to the Virgin Islands Department of Health (VIDOH) and the CDC Dengue Branch in Puerto Rico. Four of 12 patient specimens sent to the CDC Dengue Branch for diagnostic testing were confirmed as dengue. Although VIDOH had observed an increase in passive dengue reporting, reliable baseline case counts were unavailable for comparison. An investigation was begun to determine the incidence of recent dengue virus (DENV) infection in schools and islandwide.

A seroincidence study was conducted at six schools in addition to the index school, using a stratified two-stage cluster sampling methodology. Of 320 participants, 40 (20%) of 203 students and 20 (17%) of 118 staff members were immunoglobulin M (IgM) anti-DENV positive, indicating DENV infection within the preceding 3 months. Four students were polymerase chain reaction–positive for DENV-1 or DENV-4, indicating viremia and current infection.

Environmental sampling was conducted at the seven study schools and three additional schools to determine the presence of adult and immature mosquitoes. Containers with immature mosquitoes were found at all schools, and adult *Aedes aegypti* mosquitoes, the primary vector for dengue in St. Croix, were found in all but one school.

Retrospective case finding for suspected dengue cases was performed at St. Croix’s only hospital by looking for patients tested for IgM anti-DENV in 2012. Of 194 tests performed, 61 (31%) were IgM anti-DENV positive, and 42 (22%) were reported to VIDOH, with an average delay of 20 days (range: 0–47 days) after testing; 152 (78%) of the 194 tests were performed in November–December. On average, patients with dengue seek medical attention on day 4 after illness onset (2), but IgM anti-DENV is only detected by the test kit in 58% of patients at this time (Dengue Branch, Division of Vector-Borne Diseases, National Center for Emerging and Zoonotic Infectious Diseases, CDC, unpublished data, 2012). These findings highlight the need to use a combination of molecular diagnostics and immunodiagnostics to diagnose dengue early in the course of the illness (3). Initiatives to enhance dengue surveillance and reporting are ongoing at VIDOH.

This investigation suggested that students and school staff members were part of a larger islandwide dengue outbreak that might not have been identified without school reporting because only a small proportion of suspected cases were reported to VIDOH. However, because both *A. aegypti* and viremic students were detected in schools, transmission in this environment could not be excluded.

U.S. Virgin Islands residents should undertake practices to reduce mosquito production around dwellings and schools, and residents and visitors should take personal protective measures to avoid mosquito bites.* Health-care personnel in the United States should remain alert for dengue in travelers returning from the Caribbean, the most common regional source for dengue imported into the United States by travelers (4).

## Footnotes

* Additional information available at http://www.cdc.gov/dengue/prevention.
